# Household-Level Determinants of Soil and Water Conservation Adoption Phases: Evidence from North-Western Ethiopian Highlands

**DOI:** 10.1007/s00267-015-0635-5

**Published:** 2015-12-08

**Authors:** Akalu Teshome, Jan de Graaff, Menale Kassie

**Affiliations:** Soil Physics and Land Management Group, University of Wageningen, P.O. Box 47, 6700 AA Wageningen, The Netherlands; Amhara Regional Agricultural Research Institute, P.O. Box +527, Bahir Dar, Ethiopia; International Maize and Wheat Improvement Centre (CIMMYT), P. O. Box 1041, Village Market, Nairobi, 00621 Kenya

**Keywords:** Adoption phases, Soil and water conservation, Ordered probit, Ethiopia, Africa

## Abstract

Soil and water conservation (SWC) practices have been promoted in the highlands of Ethiopia during the last four decades. However, the level of adoption of SWC practices varies greatly. This paper examines the drivers of different stages of adoption of SWC technologies in the north-western highlands of Ethiopia. This study is based on a detailed farm survey among 298 households in three watersheds. Simple descriptive statistics were applied to analyze the stages of adoption. An ordered probit model was used to analyze the drivers of different stages of adoption of SWC. This model is used to analyze more than two outcomes of an ordinal dependent variable. The results indicate that sampled households are found in different phases of adoption, i.e., dis-adoption/non-adoption (18.5 %), initial adoption (30.5 %), actual adoption (20.1 %), and final adoption (30.9 %). The results of the ordered probit model show that some socio-economic and institutional factors affect the adoption phases of SWC differently. Farm labor, parcel size, ownership of tools, training in SWC, presence of SWC program, social capital (e.g., cooperation with adjacent farm owners), labor sharing scheme, and perception of erosion problem have a significant positive influence on actual and final adoption phases of SWC. In addition, the final adoption phase of SWC is positively associated with tenure security, cultivated land sizes, parcel slope, and perception on SWC profitability. Policy makers should take into consideration factors affecting (continued) adoption of SWC such as profitability, tenure security, social capital, technical support, and resource endowments (e.g., tools and labor) when designing and implementing SWC policies and programs.

## Introduction

### Background and Research Objective

The Ethiopian economy is heavily dependent on agriculture which is dominated by subsistence smallholder farmers that are partially integrated into markets. The fate of the agricultural sector directly affects economic development, food security, and poverty alleviation. However, the role of this sector in alleviating poverty and food insecurity is undermined by land degradation such as soil erosion and nutrient depletion (Bekele and Drake 2003; Taddese 2001; Tekle 1999).

Over the last four decades, the government of Ethiopia and a consortium of donors have been promoting soil and water conservation (SWC) technologies for improving agricultural productivity, household food security, and rural livelihoods, while simultaneously mitigating environmental degradation. Smallholders’ agriculture in the country is nonetheless characterized by widespread failure to make adequate SWC and soil replenishment investments in order to sustain the productivity of farmlands (Shiferaw and Holden 1998, 1999; Bewket 2007; Tefera and Sterk 2010; Kassie et al. 2010). In some cases, farmers have dis-adopted (abandoned) earlier adopted technologies (Shiferaw and Holden 1998; Tadesse and Kassa 2004; BoARD 2010). Moreover, farmers also modify or adapt the technology to their own real situations, among others by reducing the area occupied by SWC line interventions (e.g., soil bunds or stone bunds along the contour lines to reduce soil erosion).

A better understanding of constraints that condition farmers’ adoption behavior is therefore important for designing promising pro-poor policies that could stimulate and sustain adoption of SWC and agricultural productivity. A substantial literature has explored the adoption process of SWC technologies in order to understand the failure to make these critical investments (Ervin and Ervin 1982). Most previous adoption studies in Ethiopia and elsewhere (e.g., Tesfaye et al. 2013; Kassie et al. 2009; Tiwari et al. 2008; Bewket 2007; Shiferaw and Holden 1998) assumed homogenous adopters (all adopters are at the same stage) while farmers are at different stages of adoption. Adoption analyses made without considering the different stages of adoption in a complex farming system may underestimate or overestimate the influences of various factors on the decision to adopt. Like other technologies farmers pass through different stages in adopting SWC measures and also these measures are long-term investments which require continuous maintenance. This suggests that it is important to understand the different adoption phases (dis-adoption/non-adoption, initial adoption, actual adoption, and final adoption; defined in Table 1) instead of focusing on binary adoption decision. In this paper, we study the main institutional, socio-economic, and bio-physical drivers for the different stages of adoption of SWC technologies in three watershed areas of north-western Ethiopian highlands. The SWC technologies considered in this study include soil bunds, *Fanya juu* bunds (made by digging a trench and throwing the soil uphill to form an embankment), and stone bunds.^1^

**Table 1:** Soil and water conservation adoption phases and their indicators

Categories	Indicators
Dis-adopters/non-adopters	Abandoned the SWC measures and/or never used SWC measures on any of their plots
Initial adopters	Established SWC line interventions on up to 25 % of sloping farm land (experimentation phase) and did not yet expand them to other plots
Actual adopters	Established and maintained the initial SWC measures during past 4 years, and started to expand them on at least 26–50 % of the vulnerable farm land
Final adopters	Continued use, expanded, and more than 5 years maintained on their own motivation, and in total covering 51–100 % of sloping farm area

### Soil and Water Conservation Practices in Ethiopia

The importance of soil conservation was largely neglected in Ethiopia prior to 1974. The problem attracted the attention of policy makers and international donors only after the disastrous drought and famine of 1974. An effort to halt the problem of soil erosion started after the Ethiopian government initiated massive soil conservation programs following the 1975 land reform. A large number of conservation and afforestation projects were undertaken by food-for-work (FFW) programs (Hurni 1988). This massive campaign in soil conservation under FFW did not bring a wide dissemination and adoption of the practices by farmers. This is because farmers constructed SWC practices during the campaign, but they had no interest to implement or expand these without food for work (Shiferaw and Holden 1998). Most of the conservation measures were removed after the government changed in 1991 (Shiferaw and Holden 1998).

Between 1995 and 2009, soil conservation activities have been undertaken as part of the agricultural extension package of the present government through mass mobilization with a top-down approach and without incentives for the time farmers spent on SWC activities. The approach was to construct conservation measures at individual level but not at watershed level. Emphasis was given to the quantity of measures rather than the quality of measures. SWC is mainly limited to physical measures. Dis-adoption and non-adoption of SWC measures were common phenomena in this period. This indicates that the extension system did not bring about behavioral changes among farmers probably because the focus was on changing the farmland rather than farmers’ behavior.

Since 2010, the government of Ethiopia has embarked again on a massive SWC campaign. The current approach is also mass mobilization, but then at watershed level. And there is an attempt to make such SWC program more participatory. In each watershed area, agricultural offices along with local administrators organize a 15-day farmers’ workshop to create awareness about the problems of soil erosion and its causes. During the workshop, farmers prioritize their major natural resource problems, causes, and possible solutions. Then, they reach consensus about the natural resource problems that require collective action. Farmers participate in SWC activities in nearby sub-watershed areas.

## Conceptual Framework

**Fig. 1:**
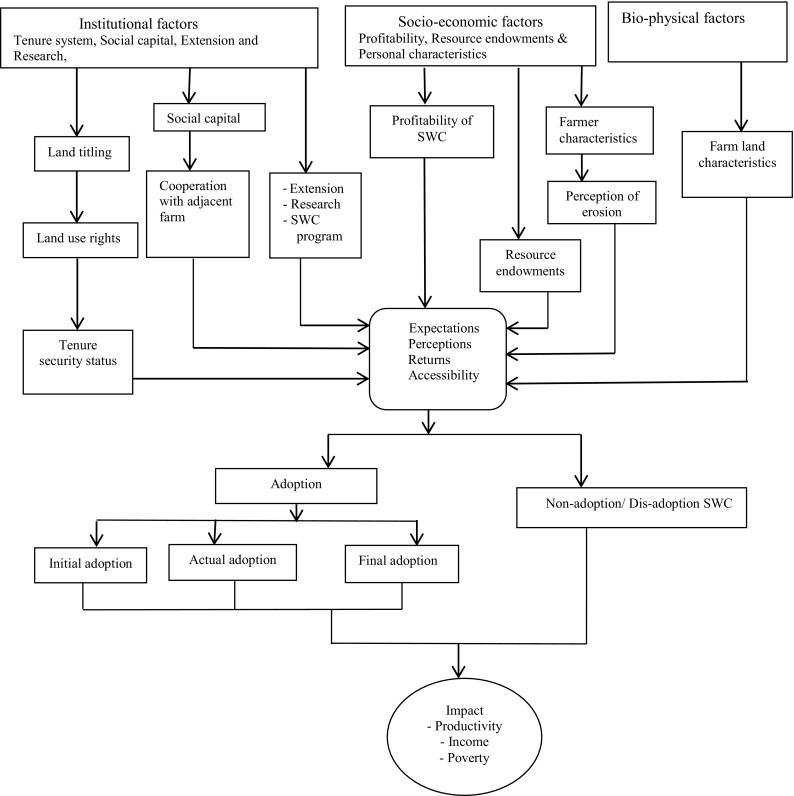
Conceptual framework of the institutional, socio-economic, and bio-physical aspects of the adoption phases

Our conceptual framework is based on the adoption process of investment in SWC measures (de Graaff et al. 2008) and on the concept of dis-adoption (abandonment) of the earlier adopted technologies (Neill and Lee 2001). This framework also incorporates important elements from decision-making processes for the use of soil conservation practices (Ervin and Ervin 1982), property rights and investment incentives (Besley 1995), and the role of social capital (Foster and Rosenzweig 1995; Nyangena 2008; Njuki et al. 2008). This analytic framework includes all major institutional and socio-economic aspects of SWC (Fig. 1).

Adoption is a sequential decision process and one could distinguish three major phases, i.e., the acceptance phase, the actual adoption phase, and the continued use phase (de Graaff et al. 2008). The acceptance phase generally includes the awareness, evaluation, and the trial stages and eventually leads to starting investment in certain measures. We will refer to this as the initial adoption phase, which is basically a pilot phase in which farmers experiment with SWC measures. The actual adoption phase is a stage whereby SWC investments are already made on part of the land since a few years, on more than a trial basis. The third phase, final adoption, is a stage in which the existing SWC measures are maintained over many years and farmers are intrinsically motivated to expand these measures to untreated plots. In addition, some farmers may dis-adopt (or abandon) once-adopted technologies, while some farmers will not adopt SWC measures at all for various reasons. Therefore, there are four major categories in the adoption process as defined below: initial adopters, actual adopters, final adopters, and non-adopters/dis-adopters^2^ (Table 1).

## Methodology

### Description of Study Areas

**Fig. 2:**
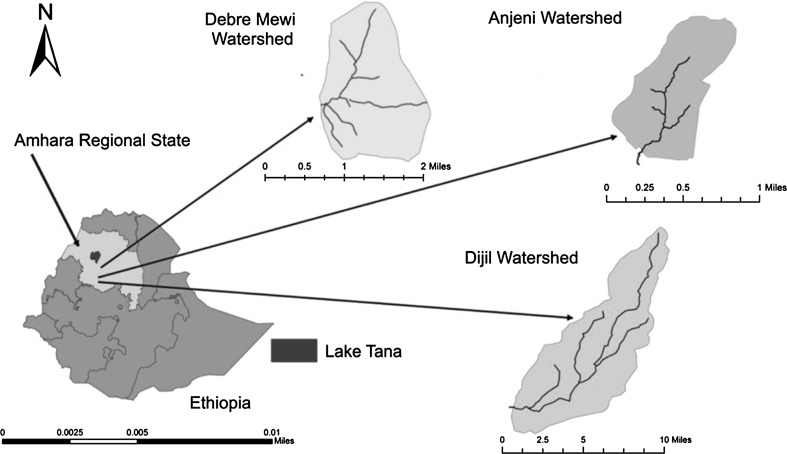
Map of study areas

The study was undertaken in three selected watersheds (Debre Mewi, Anjeni, and Dijil watersheds) of East and West Gojam Zones of Amhara Region, Ethiopia (Fig. 2). These watersheds are part of the north-western highlands of Ethiopia. The zones and the watersheds are selected purposively because of their specific experience with SWC development activities, and they differ in the extent of SWC measures that have actually been implemented. Moreover, the watersheds have diverse physical and socio-economic characteristics (Table 2). Agricultural systems in these watersheds are small-scale subsistence crop–livestock mixed farming systems.

**Table 2:** Socio-economic and physical characteristics of the study areas

Features	Anjeni watershed	Dijil watershed	Debre Mewi watershed
Altitude (m.a.s.l)	2450	2480	2300
Average annual rainfall (mm)	1790	1300	1260
Dominant soil types	Alisols, Nitosols, Regosols, and Leptosols	Nitosols	Vertic Nitosols, Nitosols, and Vertisols
Degradation	Degraded	Very degraded	Not heavily degraded
Dominant crop in farming systems	Barley	Oats	Tef
Average number of TLU (tropical livestock units) per farm	5.2	6.0	4.6
Productivity	Low	Low	High
Number of households	95	628	324
All weather road and transport access	Poor	Good	Good
Availability of local market	Yes	No	Yes
Distance to district market (Km)	20	8	12
SWC projects (exposure to SWC)	SCRP (long term)	SIDA; SLM-GIZ	No specific project

Source: Aemro 2011; Tesfaye 2011; Zegeye 2009; Zeleke and Hurni 2001; own surveys

#### Anjeni Watershed

This watershed is situated in Dembecha district of West Gojam Zone at 260 km southeast of Bahir Dar. Anjeni lies at 10.68°N and 37.53°E at an altitude of approximately 2450 m.a.s.l. The watershed covers an area of 113 ha. It is home to 95 households. Anjeni receives an average annual rainfall of around 1790 mm. The crops grown are barley, tef, maize, wheat, faba bean, potato, niger seed (*Guizotia abyssinica*), field pea, lupine, and linseed.

Soil and water conservation measures have a long history in this watershed. *Fanya juu* soil conservation bunds were introduced in 1984 by the soil and water conservation project (SCRP) which was initiated by Bern University of Switzerland in collaboration with the Ethiopian Ministry of Agriculture. The construction of bunds was done by local communities without payment for individual participating farmers. As an incentive, a health clinic was constructed by the project to compensate for labor and material contributions by the community. The Anjeni watershed has been one of the six SWC research experimental stations of the country. Moreover, SWC measures have also been disseminated in the watershed by the government extension program.

#### Digil Watershed

The Digil watershed is found in Gozamen district of East Gojam Zone. It is located at 10.24°N and 37.43°E at an altitude of approximately 2480 m.a.s.l. and 285 km southwest of Bahir Dar. Dijil watershed (Melit, Enerata, Yaya, and Yedenigia villages) covers an area of 936 ha. The total number of households in the watershed is 628. The average annual rainfall of the watershed is 1,300 mm. The major crops grown in the watershed are oats, wheat, tef, barley, faba bean, and potato.

There was an attempt to introduce soil and water conservation measures in the mid-90s in Dijil watershed areas with the regular extension program. Rigorous SWC activities were implemented specifically in Melit village in 1999 by the District Agriculture Office with financial support from the Swedish International Development Agency (SIDA) as part of its on-farm research program in Amhara region. The conservation measures such as soil, stone, and *Fanya juu* bunds were introduced by the project. Moreover, along with the SWC structures (*Fanya juu* and soil bunds) multipurpose trees like Sesbania, Grevillea, and different Acacia trees were planted for stabilizing the structures. Currently, different NGOs are involved in SWC activities in the area, such as SLM-GIZ (The German Society for International Cooperation) and Megibare Senay.

#### Debre Mewi Watershed

This watershed is located in Yilmana Densa and Bahir Dar Zuria districts of West Gojam Zone. It is located at 11.34°N and 37.43°E. It is situated at an altitude of about 2300 m.a.s.l. and receives an average annual rainfall of about 1260 mm. The total area of the watershed is estimated at 523 ha, and about 324 households are living in the watershed. Major crops grown in the watershed are tef, maize, barley, finger millet, wheat, faba bean, potato, grass pea, and noug (*Guizotia abyssinica*).

In Debre Mewi areas, SWC measures were introduced in 1990s with the regular government extension program. Different approaches have been followed to disseminate SWC practices in the area. Before 2005, farmers were participating in SWC programs through mass mobilization with a top-down approach. The approach was not watershed based but it was Kebele based (the lowest administrative body). Thus, farmers constructed bunds for other villages (at distance) in their Kebele. Some of the farmers were not the beneficiaries of what they constructed. They constructed bunds without incentives. Between 2005 and 2009, individual-level implementation rather than mass mobilization of SWC was advocated. However, it was also not effective. Currently, the watershed approach is being used again through community mass mobilization. There is an SWC experimental site in the Debre Mewi area which is handled by Adet Research Centre in collaboration with the SWHISA (Sustainable Water Harvesting and Institutional Strengthening in Amhara) project since 2008. Adet Research Centre and the District Agricultural Office are involved in the dissemination of SWC measures in the area. Although Debre Mewi is a high-production area, soil erosion is now a severe problem. Currently, gully erosion is threatening cultivated and grazing land in the watershed. The common type of physical SWC measure introduced in the area is soil bunds, and very recently also *Fanya juu* bunds.

### Sampling and Data Collection

The data for this study were obtained from 298 farm households surveyed in the three watersheds in 2011 (actually the data were collected from 300 households, but two households could not recall the years on which they constructed their bunds). The survey was conducted on a one-to-one interview basis using a structured survey questionnaire. A pre-test survey was also conducted in order to customize the questionnaire more to the situation in each study site.

The enumerators were supervised by the first author of the paper. The first author was in the field with the enumerators throughout the data collection period. The same person also participated in data collection and conducted group discussions to collect general information related to SWC and the watersheds.

In the first stage of the sampling procedure, the watersheds were selected purposely based on their SWC experience. In the second stage, farmers from each watershed were selected randomly from lists of all households in the watershed. A total of 60, 125, and 113 farmers were selected randomly from Anjeni, Dijil, and Debre Mewi watersheds, respectively. We used a formula for selecting sample size, with farm size and variation in farm size as determining factors. We found that the farm size in Digil and Debre Mewi appeared to be more skewed and therefore required a larger sample. The coefficient of variation (CV) of farm size is 0.77, 1.11, and 1.06 for Anjeni, Dijil and Debre Mewi, respectively.

The survey collected detailed information about household characteristics and labor resources, institutions and social capital, household assets, land resources and plot characteristics, and soil and water conservation (SWC) investments (see Table 3 below).

**Table 3:** Description and summary statistics of the variables used in the analysis

Variables	Description	Mean	SD
Household characteristics and labor resources			
Age	Age of household heads (in years)	45.35	12.34
Family size	Size of households (in numbers)	5.80	1.77
Farm labor	Persons working fulltime in agriculture (it includes the hired laborer on annual base);(in numbers)	2.18	0.70
Distance from road	Distance to main road from home (in walking minutes)	15.00	13.33
Land resources			
Average parcel size	Average parcel size (total farm size divided by the number of parcels); (in ha)	0.26	0.62
Cultivated land size	Actual cultivated land size (it refers to the annual crop production area); (in ha)	1.03	0.50
Farm size	Total area of farm (cultivated land, grazing land, woodland, and bare land); (in ha)	1.17	0.53
Flat slope	Flat slope (1 if on average the slopes of parcels are flat (<10 %), 0 otherwise)	0.101	–
Moderate Slope	Moderate slope (1 if on average the slopes of parcels are moderate steep (between 10 and 20 %), 0 otherwise)	0.515	–
Steep slope	Steep slope (1 if on average the slopes of parcels are steep (>20 %), 0 otherwise)	0.383	–
Other resources			
Size of iron roof^1^	Size of iron roof house (number of iron sheets)	55.54	20.72
Tools	Ownership of tools (1 if the household has tools (e.g., shovels), 0 otherwise)	0.596	–
Off-farm income	Average off-farm monthly income (in Birr^2^)	56.72	147.29
Institutions and social capital			
Tenure security	Perception of tenure security (1 if feeling secure, 0 otherwise)	0.802	–
SWC training	Training in SWC measures (1 if the household got training on SWC, 0 otherwise)	0.361	–
SWC program	Presence of SWC assisted program in the village/watershed in past/present (1 if there is an SWC program, 0 otherwise)	0.547	–
Low cooperation	Low cooperation with adjacent farm (1 if the extent of working together between adjacent farms in erosion control is low, 0 otherwise)	0.243	–
Medium cooperation	Medium cooperation with adjacent farm (1 if the extent of working together between adjacent farms in erosion control is medium, 0 otherwise)	0.291	–
High cooperation	High cooperation with adjacent farm (1 if the extent of working together between adjacent farms in erosion control is high, 0 otherwise)	0.465	–
Formal position	Executive bodies in formal associations (1 if the household has position in executive body, 0 otherwise)	0.088	–
Labor sharing (assistance)	The number of participating farmers during labor-sharing activities (labor shortage periods like weeding and harvesting); (numbers)	5.82	6.91
Perceptions			
Erosion problems	Perception on erosion problem (1 if erosion is perceived, 0 otherwise)	0.969	–
SWC profitability	Perception on the profitability of SWC (1 if profitability is perceived, 0 otherwise)	0.979	–

^1^Proxy variable for wealth. The size of an iron sheet is 2 × 0.75 m. The total size of an iron roof does not indicate the house size of the farm household. It includes the veranda. The roofing design also affects the size of iron roof vis-a-vis the house size

^2^Birr is the unit of Ethiopian currency. It is equal to 0.059 Dollar (2011)

### Analytical Model

Some multinomial choice variables are inherently ordered, for example the adoption phases of SWC measures (non-adoption/dis-adoption, initial adoption, actual adoption, and final adoption). In this case, although the outcome is discrete, the multinomial logit or probit model would fail to account for the ordinal nature of the dependent variable. The use of the ordered probit is appropriate when the dependent variable involves more than two alternatives that must take a logical ordering form as it is in our case.

Following Greene (2003), the ordered probit model can be determined by

1 $$\begin{array}{*{20}c} {y_{i}^{*} = X^{\prime}_{i} \beta+ \varepsilon } & {i = 1, \ldots N{\text{farmer}}} \\ \end{array},$$

where $i$ refers to the observation (i.e., a farmer), $y_{i}^{*}$ is a latent variable (i.e., unobservable) that represents the adoption phases of farmer $i$, $X_{i}$ is a vector of socio-economic and institutional variables including a constant, $\beta$ is a vector of parameters to be estimated, and $\varepsilon$ are the random error terms assumed to be standard normal distributed. Since $y_{i}^{*}$ is latent (unobserved), we observe discrete responses of the variable $y_{i}$ as follows:

2 $$y_{i} = 0\,({\text{dis}}\, ( {\text{non){-}adopters}})\,if\,y_{i}^{*} \le 0 ,$$

3 $$y_{i} = \,1\,({\text{initial}}\,{\text{adopters}})\,if\,0 < y_{i}^{*} \le \mu_{1} ,$$

4 $$y_{i} = 2\,({\text{actual}}\,{\text{adopters}})\,if\,\mu_{1} < y_{i}^{*} \le \mu_{2} ,$$

5 $$y_{i} = 3\,({\text{final}}\,{\text{adopters}})\,\,if\,\mu_{2} < y_{i}^{*} \le \mu_{3}.$$

The $\mu_{j}$ s are unknown ordered threshold parameters to be estimated with the unknown coefficients $\beta$. The probability that the ordered dependent variable $y$ takes the different possible values is

6 $${\text{prob}}\,(y = 0/X) = \phi \,( - X^{'} \beta ) ,$$

7 $${\text{prob}}\,(y = 1/X) = \phi \,(\mu_{1} - X^{'} \beta ) - \phi \,( - X^{'} \beta ) ,$$

8 $${\text{prob}}\,(y = 2/X) = \phi \,(\mu_{2} - X^{'} \beta ) - \phi \,(\mu_{1} - X^{'} \beta ) ,$$

9 $${\text{prob}}\,(y = 3/X) = \phi \,(\mu_{3} - X^{'} \beta ) - \phi \,(\mu_{2} - X^{'} \beta ),$$

where $\phi$ indicates a cumulative normal distribution. The cut-points $\mu_{j}$ divide the categories of the dependent variable.

The marginal effect is used to determine the influences of the independent variable per unit change on the dependent variable while everything else is constant. Computation of marginal effects is meaningful for the ordered probit model because estimated parameter coefficients do not represent the magnitudes of the effects of independent variables on the categories of dependent variable. Therefore, the marginal effects of changes in the regressors are

10 $$\frac{{\partial \,{\text{prob}}\,(y = 0/X)}}{\partial X} = - \phi \,(X^{'} \beta )\beta ,$$

11 $$\frac{{\partial \,{\text{prob}}\,(y = 1/X)}}{\partial X} = [\phi \,( - X^{'} \beta ) - \phi \,(\mu_{1} - X^{'} \beta )]\beta ,$$

12 $$\frac{{\partial \,{\text{prob}}\,(y = 2/X)}}{\partial X} = [\phi \,(\mu_{1} - X^{'} \beta ) - \phi \,(\mu_{2} - X^{'} \beta )]\beta ,$$

13 $$\frac{{\partial \,{\text{prob}}\,(y = 3/X)}}{\partial X} = [\phi \,(\mu_{2} - X^{'} \beta ) - \phi\, (\mu_{3} - X^{'} \beta )]\beta .$$

The parameter of the ordered probit model is estimated by the maximum likelihood method. We report the marginal effects of the variables.

### Review of Major Variables

Adoption phases (dependent variables) are categorized in this study based on the extent of implementation of SWC measures on the farms and the age of SWC measures (Table 1). The key socio-economic and institutional variables expected to influence the investments in SWC measures that were investigated in this study are household characteristics and labor resources, land and other resources, institutions and social capital, and perceptions of farmers (Table 3). Some of these variables are briefly discussed below.

The establishment and maintenance of SWC measures is labor intensive. Consequently, the availability of farm labor at the household level affects the adoption of SWC (Neill and Lee 2001). Households with a large amount of farm labor are probably better able to provide the labor required for the construction and maintenance of SWC measures (Tenge et al. 2005).

SWC investments are determined by qualities and quantities of land resources (Amsalu and de Graaff 2007). The average parcel size provides an indication of the fragmentation of farm land. Land fragmentation may weaken farmer’s interest and motivation for investing in SWC practices. In addition, the total cultivated land, which refers to the annual crop production area, can influence investment in SWC as well. This is because the opportunity cost^3^ of the cultivated land lost due to the width of conservation measures may be greater than the benefits of SWC structures especially for small farmers (Hengsdijk et al. 2005).

Tenure security influences the propensity to invest in SWC (Gebremedhin and Swinton 2003). Tenure security measures the perception of not running the risk of losing land at some time in the future. Investment is undertaken when the household is assured that it will reap the benefits for a considerable time period. In addition, project-supported SWC intervention programs are likely to influence the adoption of SWC measures (Posthumus et al. 2010). Project-supported interventions generally have ample resources and incentives for SWC.

Investment behavior of farmers is also shaped by the level and type of social capital (Nyangena 2008). This is because the social capital/social network influences farmers’ collaboration, preferences, transaction costs, and information exchange (Grootaert et al. 2004; Läpple and Rensburg 2011). Continued use of SWC measures is influenced by the cooperation and willingness of the adjacent farm owners to (construct and) maintain SWC measures. This is because there is a strong physical interdependency between adjacent farms with respect to hydrology and soil erosion. This aspect highlights the social components of SWC measures. The availability of labor through labor sharing (assistance) gives a chance to relax labor constraints of SWC investments (Mbaga-Semgalawe and Folmer 2000).

The perception of the economic significance of soil erosion and SWC is also important for the adoption of SWC measures. Farmers’ decisions pertaining to SWC are largely determined by their knowledge of the problems (Amsalu and de Graaff 2006). Moreover, the perception of the marginal net benefits must be greater than the marginal cost of SWC investment in order to undertake and maintain the SWC investment.

## Empirical Results

### Data and Descriptive Statistics

The results of the descriptive analysis indicate that sample households are found in different stages of adoption (Table 4). Among the sample households, initial adopters (30.5 %) and final adopters (30.9 %) form the largest groups. Most of the initial adopters have implemented SWC measures in the last 2 years. About 21.2 and 18.5 % of the sample households fall under actual adopter and non-adopter/dis-adopter categories. Reasons for dis-adoption (according to farmers) are as follows: measures were built by mass mobilization without farmers’ willingness, lack of cooperation with adjacent farm owners (low social capital), free grazing, difficult for oxen plowing, and reduction of cultivable land.

**Table 4:** Distribution of SWC adopters by watershed

Adoption phase	Watersheds						Total	
	Anjeni		Dijil		Debre Mewi			
	*N*	%	*N*	%	*N*	%	*N*	%
Dis-adopter/non-adopter	0	0	11	8.8	44	38.9	55	18.5
Initial adopter	0	0	49	39.2	42	37.2	91	30.5
Actual adopter	1	1.7	42	33.6	17	15.0	60	20.1
Final adopter	59	98.3	23	18.4	10	8.8	92	30.9
Total	60	100	125	100	113	100	298	100.0

Information on the adoption process of SWC technologies in Debre Mewi watershed was gathered during an interview which was held with a key informant (the chairman of the farmers’ research group). The practice of stone and soil bunds’ construction was first introduced in the watershed in 1996–97. According to the chairman, the first attempts were based on campaign work and did neither aim at nor succeed in raising awareness about the problem of soil erosion. People were forced to partake in these programs. As a consequence, some farmers refused to implement introduced SWC measures on their farms. The chairman further claimed that today all farmers see the different types of terraces as being beneficial for their production. However, they supposedly fail to construct them because of a lack of tools, limited technical assistance, free grazing, and a lack of cooperation between farmers of neighboring fields.

The high percentage of farmers (81.6 %) involved in SWC does not mean that so much land is protected with SWC measures. There is a large difference in the intensity of SWC adoption among adopter categories.

There is also a large difference among the adopter categories across the watersheds. Almost all households in Anjeni are already final adopters of SWC practices. This is the result of long-term SWC project interventions in the area. In addition, bio-physical factors and social capital may have influenced the adoption of SWC measures. On the other hand, more non-adopters/dis-adopters are found in the Debre Mewi watershed. This is probably because of limited project-assisted SWC interventions compared to other watersheds. This could also be explained by the watershed-level physical factors to invest in conservation practices (e.g., degradation level and rainfall amount). High percentages of initial adopters are also found in Debre Mewi and Dijil watersheds.

Table 5 shows the unconditional mean analysis of the socio-economic and institutional factors determining the different categories of adoption. The F-test analysis shows significant differences among the four adopter categories for age of the household heads, average parcel size, size of cultivated land, parcels’ slope, and the number of farmers participating in labor sharing (assistance). There are no significant differences among the adopter categories in amount of farm labor, distance from road, total farm size, size of corrugated roof houses, and off-farm income.

**Table 5:** Descriptive statistics of the explanatory variables for the adoption of SWC line interventions

Variables	Mean/percentages proportion of adopter category				F/*χ* ^2^ value^a^
	Initial adopter (*N* = 91)	Actual adopter (*N* = 60)	Final adopter (*N* = 92)	Non-adopter/dis-adopter (*N* = 55)	
Household characteristics and labor resources					
Age	43.51	43.28	45.55	50.32	0.005***
Family size	5.79	5.83	5.98	5.58	0.606
Farm labor	2.14	2.18	2.30	2.03	0.141
Distance from road	13.57	14.26	16.96	15.23	0.362
Land resources					
Average parcel size	0.19	0.24	0.29	0.23	0.001***
Cultivated land	0.94	1.19	1.11	0.89	0.001***
Farm size	1.08	1.27	1.20	1.15	0.157
Parcel slope					
Flat	11.0	1.7	4.3	27.3	0.001***
Medium	45.0	61.0	50.0	54.5	0.268
Steep	44.0	37.3	45.7	18.2	0.005***
Other resources					
Size of iron roof	56.7	57.1	53.3	55.1	0.640
Tools					
Yes	54.9	70.0	76.1	27.3	0.001***
Off-farm income	72.6	34.3	49.9	66.7	0.423
Institutions and social capital					
Tenure security					
Yes	81.3	88.3	81.5	67.3	0.037**
SWC training					
Yes	28.6	49.2	46.7	16.7	0.001***
SWC program					
Yes	41.2	70.0	85.9	9.1	0.001***
Cooperation					
High	40.4	42.4	67.0	26.4	0.001***
Medium	24.7	33.9	26.4	35.8	0.394
Low	34.8	23.7	6.6	35.8	0.001***
Formal position					
Yes	13.2	6.8	9.9	1.9	0.119
Labor sharing (assistance)	5.9	6.1	7.1	3.4	0.022**
Perceptions					
Erosion problems					
Yes	95.6	100	100	90.9	0.007***
SWC profitability					
Yes	97.8	96.7	100	96.3	0.361

*, **, *** Significant at 10, 5, and 1 % level of significance, respectively

^a^F-test is used to compare the means’ difference of more than two continuous variables, but *χ* ^2^ test is used to measure an association between discrete variables

The average age of non-adopters and dis-adopters is higher compared to the other categories. On the other hand, the average size of total cultivated land of dis-adopters/non-adopters is somewhat smaller than the other categories. This might affect adoption of physical SWC measures, since it involves some loss of cultivable land. On the other hand, initial adopters, actual adopters, and final adopters have a larger number of farmers assisting in labor sharing (during the time of labor shortage, because of weddings and harvesting activities) as compared to non-adopters/dis-adopters. This indicates the importance of labor for adoption of soil and water conservation practices.

The *χ*^2^ analysis shows a significant systematic association among adopter categories in parcel slope, ownership of tools (e.g., shovels), tenure security, training in SWC, cooperation with adjacent farm owner, presence of SWC program, and perceived problems of erosion. On the other hand, there is no systematic association among adopter categories in perceived profitability of SWC and position in formal institutions.

Dis-adopters/non-adopters (27.3 %) have fewer tools (e.g., shovels) compared to other adopter categories. Initial adopters (81.3 %), actual adopters (88.3 %), and final adopters (81.5 %) feel more tenure secure than non-adopters/dis-adopters (67.3 %). This shows that households who feel a certain tenure security are more likely to invest or maintain the soil conservation measures. Moreover, initial adopters (28.7 %), actual adopters (49.2 %), and final adopters (46.7 %) have more training exposure on SWC compared to the dis-adopters/non-adopters category. Training is one means to create awareness about the problems of erosion and the benefits of SWC measures to motivate farmers to invest in SWC measures.

Non-adopters/dis-adopters are less exposed to project-assisted SWC interventions as compared to the other categories. Project-based SWC intervention may increase farmers’ ability to invest in SWC through giving incentives (tools and training). Moreover, non-adopters/dis-adopters (37.7 %) have percentage-wise less collaboration with adjacent plot owners compared to other categories of adopters. Higher percentages of initial adopters (95.6 %), actual adopters (100 %), and final adopters (100 %) perceived the problems of soil erosion compared to dis-adopters/non-adopters (90.9 %).

Although the above unconditional descriptive statistics show that there are significant differences in covariate means among the different adoption categories, a systematic rigorous analysis that considers all variables together is important to examine whether these variables have a different influence on each group of adopters.

### Results of Econometric Analysis

The results from the ordered probit models with marginal effects are presented in Table 6. The magnitude and sign of the structural coefficients allow no direct interpretation; only an increase in a variable with a positive coefficient increases the probability in the highest category (final adoption) and decreases in the lowest category (non-adoption/dis-adoption) (Greene and Henscher 2010). The marginal effects are estimated in order to provide an indication of the relative magnitude of a unit increase in the explanatory variables on the probability of being in either category. The interpretation is direct. For instance, a unit increase in parcel size will decrease the probability of non-adoption by 7 %. The signs of the marginal probability effects can only change once when moving from the smallest category to the largest one.

**Table 6:** Ordered probit results of adoption phases of SWC

Variable	Ordered probit		Marginal effects			
	Coef.	Robust std. err.	Pro (*y*_*i*_ = 0) (non-adopter)	Pro (*y*_*i*_ = 1) (initial adopter)	Pro (*y*_*i*_ = 2) (actual adopter)	Pro (*y*_*i*_ = 3) (final adopter)
Household characteristics and labor resources						
Age	−0.01	0.01	0.01	0.01	−0.01	−0.01
Family size	−0.02	0.04	0.01	0.01	−0.01	−0.01
Farm labor	0.31**	0.13	−0.05**	−0.08	0.03**	0.01*
Distance from road	0.00	0.01	−0.01	−0.01	0.01	0.01
Land resources						
Average parcel size	0.47***	0.16	−0.07***	−0.12***	0.05**	0.14***
Cultivated land size	0.16*	0.09	−0.02*	−0.04	0.02	0.05*
Farm size	−0.17*	0.09	0.02*	0.04*	−0.02*	−0.05*
Flat slope	−0.53*	0.28	0.10	0.10***	−0.07	−0.13**
Steep slope	0.08	0.16	−0.01	−0.02	0.01	0.02
Other resources						
Size of iron roof	−0.01	0.00	0.01	0.01	0.01	−0.01
Tools	0.44***	0.17	−0.07**	−0.11***	0.05**	0.13***
Off-farm income	−0.00	0.00	0.01	0.01	−0.01	−0.01
Institutions and social capital						
Tenure security	0.33*	0.19	−0.06	−0.07**	0.04	0.09*
SWC training	0.33*	0.17	−0.05**	−0.08	0.03*	0.10**
SWC program	1.15***	0.19	−0.19***	−0.24***	0.11***	0.32***
Medium cooperation	0.02	0.20	−0.01	−0.01	0.01	0.01
High cooperation	0.43**	0.18	−0.06**	−0.11*	0.04*	0.13**
Formal position	0.31	0.24	−0.04	−0.01	0.02*	0.10
Labor sharing (assistance)	0.03***	0.01	−0.01***	−0.01***	0.01***	0.01***
Perceptions						
Erosion problems	1.21***	0.41	−0.19***	−0.07	0.19***	0.21***
SWC profitability	0.56	0.34	−0.12	−0.10***	0.08	0.13**
Cut1	2.23***	0 .64				
Cut2	3.60***	0.64				
Cut3	4.38***	0.65				
Number of observations = 272 Wald *χ* ^2^ (21) = 194.48 Prob > *χ* ^2^ = 0.0000 Pseudo *R* ^2^ = 0.2489 Log pseudo-likelihood = −277.58386						

*, **, *** Significant at 10, 5, and 1 % level of significance, respectively

The highest categories (i.e., actual and final adoption phases) are discussed in detail in this paper. The results of the first and the second categories are almost the same. This is due to bell-shaped density functions of the standard normal and the logistic distribution.

The *χ*^2^ results show that likelihood ratio statistics are highly significant (*P* < 00001) suggesting that the model has a strong explanatory power. Endogeneity bias (casual relation) is suspected between tenure security and investments (initial, actual, and final adoptions). Thus, an endogeneity test is undertaken. To investigate the relationship between investment and tenure security, we used a simultaneous probit equation model which consists of two simultaneous binary choice equations. The estimation procedure comprises the following steps: First, the reduced form of tenure security (exogenous variable) is estimated and then its predicted value obtained. Second, the predicted value of tenure security is used as a regressor in the investment (all adopter categories) equation. The process is repeated for the tenure security equation using the predicted value of investment (adoption). Two-stage probit estimation results reveal that tenure security is an important factor that affects the probability of investing in soil conservation technologies. However, the reverse relation is insignificant. This shows that there is a uni-directional causal-effect relationship between investments and tenure security. The reason may be that during the previous redistribution, investments did not guarantee tenure security and most farmers have lost what they invested and were denied of their rights to compensation and payments for their investment. Investments may influence tenure security in flexible indigenous and customary land tenure systems. The same step was applied to investigate the relation between investment and ownership of tools (e.g., shovels). There is also a uni-directional causal-effect relationship between investment and ownership of tools (e.g., shovels).

### Non-adoption/Dis-adoption and Initial Adoption Phases

The study shows that the non-adoption/dis-adoption of SWC practices is higher when there is a decrease of the farm labor, average parcel size, and cultivated land size. Lack of tools and SWC training and lower degree of cooperation with adjacent farm are also the major reasons for non-adoption/dis-adoption of SWC. In addition, low level of perception about the erosion problems also contributes to non-adoption/dis-adoption of SWC practices.

The initial adoption phase is also influenced by a decrease of parcel size, lack of tools, absence of tenure security, absence of SWC program, low cooperation with adjacent farms, decreasing labor assistance, and low perception of SWC profitability.

### Actual and Final Adoption Phases

Some variables are equally important for the actual and final adoption phases of SWC. Farm labor, average parcel size, ownership of tools (e.g., shovels), training in SWC, presence of SWC assisted program, cooperation with adjacent farm owners, labor sharing (assistance), and perception of erosion problem have a positive and significant influence on actual and final adoption phases of SWC. These factors are important for a farmer to decide whether to go from the initial phase to the actual and final adoption phases.

The amount of farm labor has an influence on the actual and continued use of SWC measures. This suggests that households who have more persons fulltime involved in agriculture are more likely to invest in and maintain SWC measures. This can be explained by the fact that labor inputs constitute the largest cost factors for SWC line interventions. In addition, the average parcel size positively influences the actual and final adoption phases. The result suggests that a unit increase in parcel size results in a 5 % increase in the probability of actual adoption and a 14 % increase in the final adoption of SWC. On average, the households managed 4.5 parcels (total farm size divided by average parcel size (Table 3). Managing 4.5 parcels each at some distance from each other is cumbersome.

Ownership of tools needed for the construction of SWC measures (e.g., shovels) is found to have a significant and positive influence on actual and final adoption stages of SWC measures. The result of the marginal effect suggests that farmers who have SWC equipment are more probably to be actual (5 %) and final (13 %) adopters. This is because the availability of (conservation) tools is a prerequisite for construction and maintenance of SWC measures.

The presence of SWC assisted programs has a significantly positive influence on the actual and final adoption stages of SWC. SWC project-assisted farmers are 11 and 32 % more likely to belong to actual and final adoption phases of SWC, respectively. This shows the importance of project-assisted SWC interventions for diffusion and adoption of soil and water conservation measures. Projects generally provide training, tools, and knowledge to implement SWC measures.

Training on SWC is positively related to the actual and final adoption phases of SWC measures. The marginal effect confirms that farmers who received trainings on SWC are 3 and 10 % more likely to fall in actual and final adoption phases of SWC, respectively. Training (e.g., training delivered by the Agricultural Office) is one means to create awareness about the problems of erosion and the benefits of SWC measures to motivate farmers to invest in SWC measures.

Cooperation with adjacent farm owners in erosion control has also a positive influence on actual and final adoption stages of SWC. The result of the marginal effect indicates that farmers who have a high degree of cooperation with adjacent farm owners are more likely to be actual (4 %) and final (13 %) adopters. This shows the role of social components of SWC measures and particularly the importance of cooperation and willingness of neighboring farmers for the construction of SWC measures.

In addition, the number of farmers participating in labor-sharing (assistance) scheme influences the actual and final adoption phases of SWC measures. This suggests that farmers who work together with many farmers in their labor-sharing activities (during labor shortage time) are more likely to replicate and continue the use of SWC measures. Labor sharing is a way of smoothing labor constraints through social networks in rural areas of Ethiopia. In addition, the perception of the economic significance of erosion is positively related to the actual and final adoption phases of SWC. The marginal effect shows that farmers who perceive the problem of erosion are 19 and 21 % more likely to belong to actual and final adoption phases of SWC, respectively.

### Final Adoption Phase

The study results show that the final adoption phase is specifically influenced by different factors. The effect of cultivated land size is found to be positive and significant (*P* < 0.10) on the final adoption phase of SWC. The result of the marginal effect indicates that a unit increase in cultivated land would increase the probability of the continued use of SWC measures by 5 %. This is because the potential loss of land for SWC and temporal yield decline do not constrain the adoption of SWC for large holdings.

The slope degree of parcels influences the final adoption stage of SWC measures and is statistically significant. The finding illustrates that farmers who operate on fields with gentle slope are 13 % less likely to invest, replicate, and maintain SWC technologies. This may be explained by the positive relationships between slope level and severity of soil erosion. (Amsalu and de Graaff 2007; Anley et al. 2007).

Farmers’ perceived profitability of SWC measures has a positive (*P* < 0.01) influence on the final adoption phase of SWC measures. The marginal effect indicates that farmers who perceive SWC measures to be profitable are 13 % more likely to maintain SWC measures. This is because financially viable SWC measures encourage adoption (continued use) of SWC measures.

Tenure security is positively (*P* < 0.10) related to the final adoption phase of SWC. More specifically, the result of the marginal effect shows that tenure security significantly increases the likelihood of final adoption of SWC by a margin of 9 %. This result is consistent with findings of other studies (Neill and Lee 2001; Soule et al. 2000). On the other hand, total farm size, a proxy variable of wealth, has a negative influence on the final adoption phase of SWC measures. A unit increase in the total farm size reduces the probability of maintaining SWC measures by 5 %. This is because wealthy farmers may focus on other income-generating activities and they may give less attention to SWC measures.

We made a rerun of the model excluding the dis-adopter group (i.e., only considering the non-adopter groups), and the results are presented in Appendix 1. The estimates are quite similar except cultivated land size and farm size variables.

For a robustness check, we have run multinomial logistic regression, the results of which are shown in Appendix 2. The estimates of the two models (ordered and multinomial) are almost similar.

## Discussion

As mentioned earlier, the adoption of SWC measures is a sequential process. The factors that influence (the stages of) adoption are highly context specific, which makes generalizations difficult (de Graaff et al. 2008; Lapar and Pandey 1999). Moreover, cooperation between several different types of actors is a key to successful innovation (Klerkx and Leeuwis 2009). This is because complex technologies are developed and disseminated by innovation networks (Ekboir 2003).

This study found that final adoption depends mostly on the size of a parcel, the size of cultivable land (land fragmentation), resource endowments (labor and tools), tenure security, technical support (availability of training and SWC program), perceived erosion problems, and profitability of SWC and social capital.

On average, the sample households managed 4.5 parcels (Table 3). This study shows that land fragmentation negatively influences the continued adoption of SWC, suggesting that farmers who have a smaller parcel size and/or fragmented parcels are less likely to invest in and maintain SWC measures. This is probably because it increases the transaction cost for investments, which is in line with previous findings (Gebremedhin and Swinton 2003; Tenge et al. 2004; Sklenicka et al. 2014).

The study further revealed that technical support (availability of training and SWC programs) and resource endowments (farm labor) influenced the continued use of SWC measures. This is because these interventions are knowledge and labor intensive. These results are consistent with the findings of Bekele and Drake (2003), Posthumus et al. (2010), and Adimassu et al. (2012).

Tenure security is important to undertake long-term land improvement investments (Besley 1995). Our result is consistent with findings of Neill and Lee (2001) in Northern Honduras, Gavian and Fafchamps (1996) in Nigeria, and Gebremedhin and Swinton (2003) in the Tigray region of North Ethiopia. Conversely, Holden and Yohannes (2002) revealed that tenure insecurity had in Southern Ethiopia no negative effect on long-term investment. This difference could be explained by the differences in socio-economic and land redistribution experiences between Amhara and Southern regions.

The significance of farmers’ perception of how soil erosion affects their land productivity indicates that high awareness about soil erosion problems is crucial to increase the likelihood of adoption of SWC measures. Amsalu and de Graaff (2006) and Ervin and Ervin (1982) found similar results. Perceived profitability is also important in the adoption of SWC. Bunds have an effect on crop productivity (Nyssen et al. 2007). Promoting technologies that increase the productivity and income of farmers is therefore important to speed up the adoption process. This result is consistent with findings of Cary and Wilkinson (1997) and Amsalu and de Graaff (2007).

Cooperation with adjacent farm owners in erosion control is important for the continued adoption of SWC. There is a strong physical interdependency between adjacent farms with respect to hydrology and soil erosion. This result is in line with findings of Beekman and Bulte (2012) in Burundi.

## Conclusions

A better understanding of factors affecting adoption behavior is vital for designing promising pro-poor policies that could stimulate and sustain adoption of SWC. In this study, the adoption process of SWC measures is categorized into four major phases, i.e., non-adoption/dis-adoption, initial adoption, actual adoption, and final adoption. The study indicates that sample households find themselves in different phases of adoption due to different institutional and socio-economic factors. Among other things, these findings indicate that adoption studies should not only focus on the classic comparison between adopter and non-adopter categories, but rather investigate the adoption process of SWC measures at different phases of adoption.

The study shows that the non-adoption/dis-adoption and initial adoption of SWC are mainly due to land fragmentation, lack of technical support and resource endowment, low social capital, and low level of perception of erosion problems and profitability of SWC.

The results of the study indicate that availability of labor is very important for the actual and final adoption phases of SWC. Specifically, the maintenance costs for the final adoption stage are very important. This implies that conservation structures need to be made less labor demanding by reducing the maintenance costs, i.e., by stabilizing bunds through biological measures. The study results also indicate that ownership of tools (e.g., shovels) and project assistance for SWC interventions are very important factors that affect the actual and final adoption phases of SWC. This implies that there is a need for technical support and resources (tools for SWC measures) for farmers to increase their investment capacity and know-how in order to facilitate the adoption process.

In addition, the study reveals that social capital specifically cooperation with adjacent farm owners is a key factor for the actual and final adoption phases of SWC. This means that conservation on one farm will have little spill-over impact when farm land on adjacent farm areas is not conserved. This implies that the adjacent farm owners need to work together to avert the problems of erosion. Thus, a watershed approach applied at community level is the remedy for the problems of cooperation between adjacent farms. With a watershed approach, SWC measures are implemented more comprehensively at community level. The average parcel size is also influencing the actual and final adoption stages positively. The average parcel size is an indication of the fragmentation of the farm parcels. On dispersed and fragmented small parcels, the cost of investing in SWC measures may be excessive. Either land consolidation or alternative SWC measures are important to enhance the productivity of farm land.

The final adoption phase of SWC is positively associated with cultivated land size and farmers’ perceived profitability of SWC measures. Thus, investigation of the economic efficiency of the different SWC measures under different circumstances is of paramount importance to select feasible measures. In addition, the results of the analysis show that tenure security is an important factor that affects the final adoption phase of SWC. Secure land rights increase the planning horizon of farmers to undertake long-term investments. Therefore, the land policies should provide long-term and lasting tenure security to the farmers.

The overall results of this empirical analysis indicate that institutional and socio-economic factors functioning at national, regional, watershed, village, farm, and household level play a strong role in shaping farmers’ investment behavior at the different phases of SWC adoption. Thus, policy makers should take into consideration the factors affecting adoption (continued) of SWC such as profitability, tenure security, social capital, technical support, and resource endowments (e.g., tools and labor) when designing and implementing SWC policies and programs.

## Appendix

See Tables 7 and 8.

**Table 7:** Ordered probit results of adoption phases of SWC excluding the dis-adopter group

Variable	Ordered probit		Marginal effects			
	Coef.	Robust std. err.	Pro (*y*_*i*_ = 0) (non-adopter)	Pro (*y*_*i*_ = 1) (initial adopter)	Pro (*y*_*i*_ = 2) (actual adopter)	Pro (*y*_*i*_ = 3) (final adopter)
Household characteristics and labor resources						
Age	−0.01	0.01	0.01	0.01	−0.01	−0.01
Family size	−0.02	0.04	0.01	0.01	−0.01	−0.01
Farm labor	0.37***	0.13	−0.04**	−0.11***	0.03**	0.12*
Distance from road	0.00	0.01	−0.01	−0.01	0.01	0.01
Land resources						
Average parcel size	0.51***	0.16	−0.05***	−0.14***	0.04**	0.16***
Cultivated land size	0.11	0.01	−0.01	−0.03	0.01	0.04
Farm size	−0.13	0.09	0.01	0.04	−0.01	−0.04
Flat slope	−0.62*	0.29	0.09	0.15***	−0.07	−0.16***
Steep slope	0.01	0.16	−0.01	−0.01	0.01	0.01
Other resources						
Size of iron roof	−0.01	0.00	0.01	0.01	0.01	−0.01
Tools	0.41**	0.17	−0.05**	−0.11**	0.03*	0.13**
Off-farm income	−0.00	0.00	0.01	0.01	−0.01	−0.01
Institutions and social capital						
Tenure security	0.27	0.19	−0.03	−0.08	0.27	0.08
SWC training	0.28	0.17	−0.03*	−0.08	0.02	0.09
SWC program	1.10***	0.19	−0.14***	−0.28***	0.09***	0.32***
Medium cooperation	0.08	0.20	−0.01	−0.03	0.01	0.03
High cooperation	0.45**	0.18	−0.05**	−0.13**	0.32**	0.14**
Formal position	0.31	0.24	−0.03	−0.09	0.01	0.10
Labor sharing (assistance)	0.03***	0.01	−0.01***	−0.01***	0.01**	0.01***
Perceptions						
Erosion problems	1.26***	0.41	−0.29**	−0.14**	0.19***	0.24***
SWC profitability	0.61*	0.36	−0.09	−0.14***	0.08	0.15**
Cut1	1.98***	0.64				
Cut2	3.49***	0.65				
Cut3	4.29***	0.66				
Number of observations = 258 Wald *χ* ^2^ (21) = 170.53 Prob > *χ* ^2^ = 0.0000 Pseudo *R* ^2^ = 0.2389 Log pseudo-likelihood = −261.87						

***, **, ***** Significant at 10, 5, and 1 % level of significance, respectively

**Table 8:** Multinomial logit results of adoption phases of SWC

Variable	Adopter category					
	Initial adopter		Actual adopter		Final adopter	
	Coefficient	Robust std. err.	Coefficient	Robust std. err.	Coefficient	Robust std. err.
Constant	−1.397	2.061	−21.201***	2.551	−37.760***	2.656
Household characteristics and labor resources						
Age	−0.052**	0.023	−0.071**	0.028	−0.048*	0.028
Family size	−0.065	0.147	−0.104	0.163	−0.052	0.164
Farm labor	0.411	0.369	0.541	0.443	1.056**	0.486
Distance from road	−0.021	0.018	−0.015	0.019	0.008	0.021
Land resources						
Average parcel size	−0.760	0.682	0.201	0.728	1.270*	0.742
Cultivated land size	0.274	0.242	0.775**	0.314	0.745**	0.324
Farm size	−0.122	0.283	−0.322	0.337	−0.657*	0.341
Flat slope	−0.182	0.790	−2.844**	1.158	−1.140	1.011
Steep slope	1.500**	0.592	0.566	0.652	1.131*	0.662
Other resources						
Size of iron roof	−0.005	0.011	−0.001	0.013	−0.016	0.014
Tools	1.759***	0.490	1.877***	0.588	2.265***	0.570
Off-farm income	−0.002**	0.001	−0.004***	0.001	−0.003*	0.001
Institutions and social capital						
Tenure security	1.198*	2.112	2.112***	0.722	1.625**	0.714
SWC training	0.311	0.633	0.553	0.661	0.970	0.689
SWC program	3.158***	1.081***	4.452***	1.100	4.596***	1.089
Medium cooperation	−1.356*	0.646	−1.299*	0.725	0.793	1.069
High cooperation	−0.320	0.655	−0.448	0.746	2.297**	1.038
Formal position	1.851	1.681	0.245	1.812	2.138	1.688
Labor sharing (assistance)	0.084	0.055	0.101*	0.057	0.146***	0.054
Perceptions						
Erosion problems	0.762	1.027	17.821***	1.516	16.614***	1.406
SWC profitability	1.367	1.367	1.254	1.33658	15.125***	1.571
Base category = non-adoption/dis-adoption Number of observation = 272 LR chi(88) = 293.69 Prob > *χ* ^2^ = 0.000 Pseudo *R* ^2^ = 0.37 Log likelihood = −252.38						

***, **, ***** Significant at 10, 5, and 1 % level of significance, respectively

## Footnotes

1 The three measures are not that different, they all three have the same function: line interventions along the contour lines to reduce soil erosion.

2 It would have been good to separate non-adoption and dis-adoption groups; however, there are not enough observations that enable us to treat these two variables separately. The number of observations for dis-adopters is very small (15 observations) and not enough to independently include in the model.

3 The benefits that an individual could have received by taking an alternative action.
